# Retro-Nasal Aroma Release Is Correlated with Variations in the In-Mouth Air Cavity Volume after Empty Deglutition

**DOI:** 10.1371/journal.pone.0041276

**Published:** 2012-07-17

**Authors:** Anne Mishellany-Dutour, Alain Woda, Hélène Labouré, Pierre Bourdiol, Pauline Lachaze, Elisabeth Guichard, Gilles Feron

**Affiliations:** 1 Dental Faculty, Equipe d'Accueil 3847, Centre de Recherche en Odontologie Clinique, Clermont-Ferrand, France; 2 Centre des Sciences du Goût et de l'Alimentation, Unité Mixte de Recherche 1324 Institut National de Recherche Agronomique, Unité Mixte de Recherche 6265 Centre National de la Recherche Scientifique, Université de Bourgogne, Dijon, France; 3 Centre Hospitalo-Universitaire, Clermont-Ferrand, Service d'Odontologie, Clermont-Ferrand, France; Technical University of Dresden Medical School, Germany

## Abstract

We hypothesized that interindividual differences in motor activities during chewing and/or swallowing were determining factors for the transfer of volatile aroma from the in-mouth air cavity (IMAC) toward the olfactory mucosa. In our first experiment, we looked for changes in IMAC volume after saliva deglutition in 12 healthy subjects. The mean IMAC volume was measured after empty deglutition using an acoustic pharyngometer device. Based on the time course of the IMAC volume after swallowing, we discerned two groups of subjects. The first group displayed a small, constant IMAC volume (2.26 mL ±0.62) that corresponded to a high tongue position. The second group displayed a progressive increase in IMAC (from 6.82 mL ±2.37 to 22.82 mL ±3.04) that corresponded to a progressive lowering of the tongue to its resting position. In our second experiment, we investigated the relationship between IMAC volume changes after deglutition and the level of aroma release at the nostril. For this purpose, the release of menthone was measured at the nostril level in 25 subjects who consumed similar amounts of a mint tablet. The subjects were separated into two groups corresponding to two levels of menthone release: high (H) and low (L). The mean volume of IMAC was measured during and after empty deglutition. Group H displayed a small, constant amplitude of IMAC volume change after deglutition, while Group L displayed a progressive increase in IMAC. It is likely that Group H continuously released the aroma through the veloglossal isthmus as the mint was consumed, while Group L trapped the aroma in the oral cavity and then released it into the nasal cavity upon swallowing. These results show that the *in vivo* aroma release profile in humans depends closely on the different motor patterns at work during empty deglutition.

## Introduction

Retro-nasal olfaction, which occurs in humans while eating, is a key factor driving food perception, acceptability and intake. However, its mechanism is poorly understood. One central question arises from the repeated observation of considerable interindividual variability in the amount of aroma compounds recorded at the nostril during food consumption using atmospheric pressure ionization mass spectrometry (API-MS) [1]–[6]. The origin of this variability has not been satisfactorily explained. Recent reports have indicated that the consumption of the same amount of product by mouth can produce a 10-fold range of variation in the amount of aroma compound released at the nostril level. The same authors have reported that none of the physiological variables measured (salivary flow and composition, respiratory flow and maximum oral volume capacity) offer a solid explanation for these interindividual differences. The results of these studies ultimately suggest that chewing behavior during mastication and swallowing contribute to interindividual variations in the transfer of aroma compounds from the mouth to the nose [7]–[9].

Studies have proposed two different types of tongue-velum behaviors that transfer odorants toward the olfactory mucosa during the mastication/deglutition process. The first mechanism accounts for a relatively continuous aroma transfer profile. During mastication, respiration is maintained, though at an altered rate [10]. The intermittent elevation of the velum, particularly during expiration [11], provides a route for air passage and allows food to aggregate in the pharynx before swallowing [10], [12]. Air transfer between the oral and nasal cavities is associated with masticatory jaw movements and is measured by changes in nasal manometry values [13], [14], nasal airflow [10] and the aroma content of nasal-expired air [2]. In addition, the cyclic elevation of the velum is associated with the consumption of solid foods. In some chewing movements, the soft palate moves upward as the jaw opens and downward as the jaw closes [13], [15]. Therefore, during mastication, pulses of aromatized air may be continuously “pumped” to the nose [10]. The second mechanism accounts for a single-phase aroma transfer profile. The aromatized air is blocked by the closing of the fauces during eating, and aromatized air reaches the olfactory mucosa only after swallowing. At that time, the reopening of the sphincters is followed by a short pulse of respiration: the “swallow breath” [10], [12], [16]. This effect is reinforced by the presence of an aromatized parietal coating of foods or beverages left after deglutition [17].

Different mechanisms may explain the reportedly marked interindividual variations in aroma release profiles [2], [5], [7], [18]. The interindividual differences in tongue praxis observed during the swallowing of saliva (empty swallow) may generate different patterns of aromatized air passage through the veloglossal isthmus [19] and result in the different aroma profiles observed at the nostril level [2].

To test this hypothesis, a two-part study was undertaken. The first experiment was conducted to observe changes in the in-mouth air cavity (IMAC) volume after deglutition. The IMAC volume was measured at several time points after empty deglutition using a pharyngometer based on an acoustic echo. Two distinct modes of postdeglutition IMAC changes reflecting tongue praxis during deglutition were observed. The second experiment was then designed to test whether each mode of IMAC change was related to one of the two main aroma release profiles described in a previous study [8]. This finding would imply that the two praxis types exhibited during empty deglutition are related to the two different levels of aroma release recorded at the nostril level.

## Materials and Methods

### Ethics statement

All subjects were informed about the observational nature of these studies; they provided signed consent and received compensation for their participation. The study protocols were approved by the Comité de Protection des Personnes Est-1 (No. 2008/15; 17.04.2008) and the Direction Générale de la Santé – France (No. DGS2008-0196; 08.08.2008).

### Subjects

The first experiment involved 12 consenting Caucasian subjects (8 females and 4 males; mean age: 30±0.6 years; range: 24 to 32 years). Strict inclusion criteria were used to limit the sources of variability. All subjects were former students of the Dental Faculty of Clermont-Ferrand. The inclusion criteria were as follows: possession of healthy complete dentition, except for third molars; normal maxillomandibular relationship; no previous orthodontic treatment; no pharmacological drug that could modify salivary secretion; and no chronic pain in the temporomandibular joint and masticatory muscles. Subjects with tongue piercings or unusual swallowing and those who breathed exclusively through the mouth were excluded.

**Figure 1:**
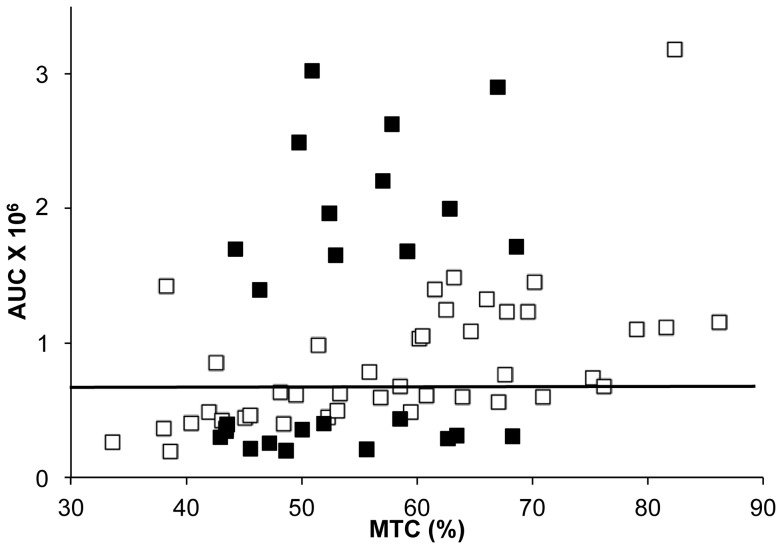
Quantity of aroma release *vs.* tablet degradation in the mouth for the 68 subjects. The total quantity of released menthone for each subject was calculated according to the area under the curve (AUC). This value was divided by the quantity of in-mouth mint tablet consumed (MTC) expressed as a percentage of the initial weight of the tablet. The AUC/MTC ratio was used to select the 25 study subjects (in black squares) from the group of 68 subjects. The objective was to obtain two highly contrasting groups of subjects. The 25 subjects were then divided into a high-releasing group consisting of 12 subjects whose values were above the median value of the group (indicated by the horizontal line) and a low-releasing group consisting of 13 subjects whose values were below the median value of the group.

The second experiment included two sessions (Table 1). The first session included 68 volunteers recruited by phone. The inclusion criteria were intentionally broad to allow a large degree of variability. The subjects participated in two one-hour measurement sessions. In the first session, the subjects were asked not to eat, drink or smoke for at least one hour before each session. The protocol used to characterize the aroma release profile has been described in detail elsewhere [8] and can be summarized as follows: the subjects were asked to put a mint tablet in their mouths and perform standardized oral food processing events for 3 minutes. The retro-nasal release of flavor from the dissolution of the mint tablet was followed by a nose-space analysis (see below). At the end of the 3 minutes, the mint tablet was weighed to measure how much the tablet had dissolved in the saliva. For the second session, 25 of the 68 subjects (9 females and 16 males; mean age: 42.9±8.9 years; range: 24 to 60 years) were selected. This selection was aimed at creating two groups of subjects who consumed similar amounts of dissolved mint tablet but had dramatically different amounts of flavor released in the nose. Therefore, the subjects who displayed the highest or lowest aroma release at the nostril were chosen from the 68 subjects who dissolved a similar amount of the mint tablet in their mouths. Based on the nasal aroma quantity released/mint tablet consumed ratio (the AUC/MTC ratio; see details below), 12 subjects were considered high-releasing (Group H), and 13 were considered low-releasing (Group L; Figure 1).

**Table 1:** Survey of the study organization.

	Session	Subjects	Calibration	Measures
First Experiment	One session	12 homogeneous subjects	Dead space Mouth length	IMAC after swallow
	First session	68 heterogeneous subjects		Aroma release Respiratory flow Salivary flow
Second Experiment		<$>\kern 25\raster="rg1"<$>		
	Second session	12 high releasing 13low releasing	Dead space Mouth length	IMAC during and after swallow

In the first experiment, strict inclusion criteria were applied to obtain a homogenous group. In contrast, the 68 subjects included in the first session of the second experiment were heterogeneous to provide two contrasting groups for the second session.

### Experimental procedures for IMAC measurements

**Figure 2:**
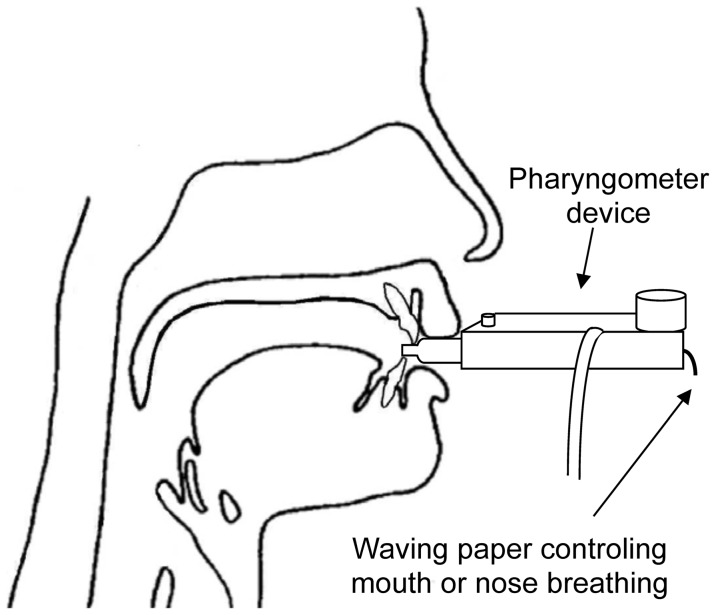
In-mouth air cavity volume evaluations with the acoustic pharyngometer.

An Eccovision® acoustic pharyngometer (Hood Laboratories, DHSS, Miami, FL, USA) was used to measure in-mouth air volumes in both experiments. The instrument consisted of four components: a wave tube, an electronics platform, a mouthpiece and a disposable filter (Figure 2). The device was linked to a control unit with signal conversion capabilities and software for data recording, processing and storing. Reflectance pharyngometry was performed with a two-microphone imaging acoustic pharyngometer. A similar apparatus was described by Louis et al. [20]. This device consisted of two microphones and one horn driver mounted to a wave tube, and it was connected to a computer with signal conversion capabilities and software for data recording, processing and storing. The pharyngometer was calibrated before recording for each patient (see below). The IMAC was measured while the subjects sat upright in a backed chair facing straight ahead. The subjects held the pharyngometer and placed their teeth against the flange of the mouthpiece. To prevent air leaks that could cause measurement errors, the subjects were told to place their lips over the flange to seal the mouthpiece (Figure 2).

In the first experiment, the IMAC volume was recorded 1, 3, 9 and 27 seconds after the swallow was triggered. In the second experiment, the IMAC volume was recorded 0, 0.2, 0.4, 0.6, 0.8, 1, 1.2, 1.4, 1.6, 3, 9 and 27 seconds after triggering the swallow. The subjects were only instructed to swallow once, at the beginning of the recording. No instruction was given for tongue position within the mouth, breathing mode (through the nose or the mouth) or interarch contact to avoid influencing the spontaneous deglutition praxis. All subjects, however, breathed through the nose during and immediately after swallowing, as indicated by the waving motion of a paper strip affixed to the entrance of the pharyngometer. Respiratory flow was only measured in Experiment 2. All IMAC measurements were repeated four times for each subject in both experiments to determine within-subject variability. The interval between successive swallows was determined by each subject. All measurements were recorded on a computer and stored for statistical analysis.

### Calibration of the pharyngometer

The oral end of the pharyngometer contained a dead volume that had to be subtracted from the IMAC values that were measured during operation. To determine the dead volume of the pharyngometer, the minimal IMAC volume value was determined by asking the subjects to breathe through their noses with their tongues against their palate and dental arches in maximal intercuspal position. Because of the dead volume, no zero values were ever recorded for the IMAC volume. Four trials were performed, and the mean was calculated. The recorded mean residual volume was subtracted from the values obtained in the experiments to obtain the real IMAC volume for each subject and each recording.

**Figure 3:**
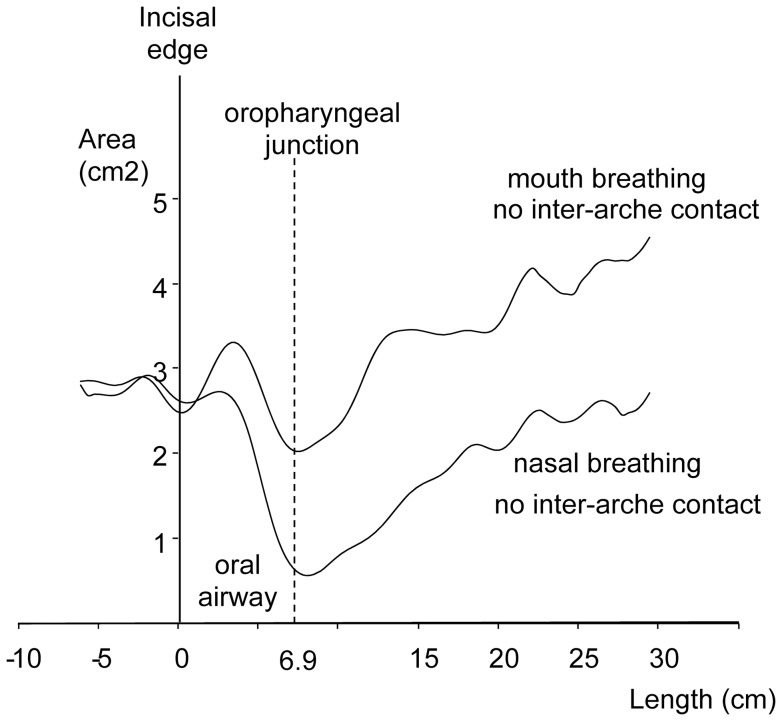
Determination of the oral airway length for the first experiment. Curves were obtained with the pharyngometer during oral or nasal breathing with the dental arches in a noncontacting position. The x-axis zero corresponds to the incisal edge.

Each subject's oral cavity length was determined to allow IMAC volume measurement with the pharyngometer. Preliminary experiments revealed that the oral cavity length was best derived from two types of IMAC volume recordings. First, the subjects breathed only through their noses without the dental arches in the contact position. Second, the subjects were asked to breathe only through their mouths (while wearing a nose-clip) and without their dental arches in the contact position (the subjects received no instruction about the tongue position in the mouth). The lowest point on the two curves depicted in Figure 3 indicates the oral cavity's posterior end. The individual oral cavity length values were then determined for each curve. The individual oral cavity length was based on the mean of four successive measurements. An intersubject mean was also calculated.

### Retro-nasal aroma release measurement

Aroma release was measured using atmospheric pressure ionization mass spectrometry (API-MS) [2]. Air from the nose was sampled from one nostril at an average flow rate of 37 mL.min^−1^ and introduced into the API-MS source of an Esquire-LC mass spectrometer ion trap (Bruker Daltonique, Wissembourg, France) through fused silica capillary tubing (i.d.  = 0.53 mm) heated to 150°C, to which a 5-kV positive ion corona pin discharge was applied [21]. Two compounds were monitored according to their protonated molecular ion (MH+): menthone (*m/z* = 155), the principal aroma compound in the mint tablet, and acetone (*m/z* = 59), which was used as an indicator of the subject's breath [4]–[6], [22], [23].

### Experimental procedures for aroma release measurement

The aroma release profile was recorded during the first session of the second experiment and monitored while the subjects consumed a commercially available mint tablet (Frisk®, Menthe Douce; Frisk International NV; *m* = 0.14 g) purchased at a local supermarket. The tablets contained a sweetener (polyols), aroma and antibinding material. The subjects were asked not to eat, drink or smoke for at least 1 hour before the session. Each subject was asked to position the plastic tube of the API-MS in one nostril and to breathe normally through the nose to control the regularity of breathing. After an initial swallow, the subjects were asked to place the tablet under their tongues, keep their lips closed throughout the measurement period and remain in a resting mode (i.e., with no mouth movements or swallowing) to prevent any connection between the mouth and the nose [1]. We opted to place the tablet under the subjects' tongues to allow the most natural and relaxed resting position possible, thus avoiding a reflex contraction of the soft palate. The subjects were then given a series of instructions to alternate three swallowing and oral movements of the tongue and jaw [8]. The signals were recorded continuously until the menthone signal reached its basal level. Three replications per subject were performed during each session. Bread and water were used as mouth cleansers between the two tests. Throughout the study, the stability of the API-MS signal was checked twice daily using a solution with a known aromatic concentration. The API-MS data were analyzed quantitatively by calculating the area under the curve (AUC) representing the total quantity of menthone released for each subject and each replication. At the end of the API-MS measurements, the subjects were asked to spit out the tablet, which was then weighed. With the initial weight predetermined, the mint tablet consumed (MTC) amount was expressed as a percentage: ([Initial weight (g) – final weight (g)]/Initial weight). The ratio was thus calculated as AUC/MTC. Respiratory flow was recorded as follows: The whole nasal airflow rate was monitored using a flow meter (Pulmo System II, MSR, Rungis, France). During the two sessions, the subjects wore a mask over the nose and were asked to breathe normally for 3 min with their mouth closed. Six one-minute records were obtained and their values averaged for the two following parameters: the breathing frequency (BF), expressed as the number of breaths per minute, and the volume of air (VT) inhaled and exhaled, expressed in liters. Next, the breath flow rate (Ls^−1^) was calculated: BFR  =  (BF*VT)/60.

### Saliva measurement

Resting (rSF) and stimulated (sSF) salivary flow rates were measured during the second experiment. Before samples were collected, each subject was asked to swallow to empty the mouth of saliva, and nonstimulated whole saliva was collected by instructing the subject to spit out saliva into a preweighed cup every 30 seconds for 5 min [24]. rSF was measured once during the two sessions. The stimulated whole saliva was collected by instructing the subject to chew a piece of Parafilm™ (0.5 g ±0.2 g) for 1 min. After 30 seconds, the subject was asked to spit out the saliva, continue chewing for another 30 seconds and then spit out both the saliva and the piece of Parafilm™. The sSF measurements were replicated three times in one session. The salivary flow rates were expressed in mL.min^−1^.

### Statistical analysis

The statistical analysis was performed using either the SPSS software (Version 11.5 for Windows, 2005; SPSS Inc., Chicago, IL, USA) or STATISTICA software, Version 10 (StatSoft, France). To check for any repetition effect, a one-way repeated-measure ANOVA was performed with the four replicates as the within-subject factor and the subjects as the between-subject factor. A repeated-measures ANOVA was then performed using the four swallowing times as the within-subject factor and the subjects as the between-subject factor. When the repeated-measures ANOVA indicated a significant difference between the times, either a Student-Newman-Keuls (SNK) test (Experiment 1) or a multivariate analysis of variance (MANOVA; Experiment 2) was conducted to compare the means. ANOVA analyses were also performed on the other variables (i.e., saliva and respiratory flows, sex and age according to group [H and L]). When there was no normality and/or variance homogeneity hypothesis, nonparametric tests (Mann-Whitney U) were applied. Values are given as the mean ± standard error of the mean (SEM), and a 5% Type I error was set.

## Results

The mean value of the oral cavity length, based on the values for the 12 subjects in the first experiment, was 6.9 cm ±0.57 (*n* = 12). The mean oral cavity length was 8.01 cm ±0.9 (*n* = 25) for the subjects in the second experiment. Repetitions had no effect on the IMAC volumes obtained at different times after swallowing in either experiment (ANOVA, *p*>0.05).

### Experiment 1

**Figure 4:**
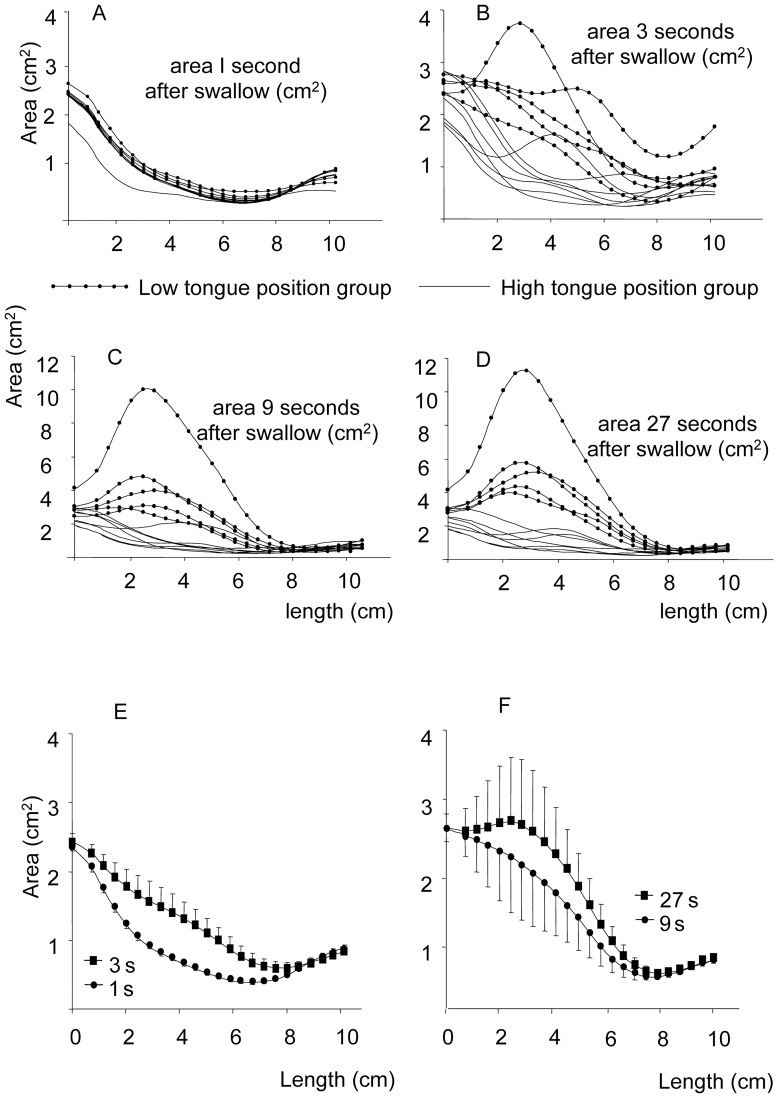
In-mouth air cavity cross-section as a function of the in-mouth air cavity, antero-posterior section level. A, B, C, D: Individual in-mouth air cavity cross-section (cm^2^) at four times points (1, 3, 9 and 27 seconds) after swallowing (*n* = 12). The two groups were identified in Experiment 1. After deglutition, one group displayed a large in-mouth air cavity (lines with dots), and another displayed a small in-mouth air cavity (lines without dots). E and F: Mean tongue position (mean cross section area ± standard error) of the 12 subjects at 1, 3, 9 and 27 s after swallowing. The subjects received no about the tongue position in the mouth, breathing or inter-arch contact. Monitoring showed that the subjects breathed through their noses.

The IMAC volumes measured 1, 3, 9 and 27 seconds after swallowing are shown in Figure 4A, B, C, D for each individual and in Figure 4E and Table 1 for the entire group (*n* = 12). The figure displays the IMAC cross-section (cm^2^) as a function of the in-mouth anteroposterior section level (cm). We observed that the IMAC was larger in the anterior part of the mouth and smaller in the posterior part near the isthmus. The IMAC volume was different at each of the four time points after swallowing (F [2,83]  = 48; *p*<0.001), and this time effect was strongly influenced by the subjects (F [25,83]  = 6; *p*<0.001; repeated-measures ANOVA). Two groups of subjects were clearly distinguished according to their tongue positions after swallowing (SNK, *p*<0.05; Figure 4A, B, C, D). Group 1 consisted of seven subjects with a high tongue position after swallowing. Group 2 consisted of five subjects with a low tongue position after swallowing. In the first group, the mean IMAC volume increased progressively (Table 2) from 6.8 mL 1 second after swallowing to 22.8 mL after 27 seconds, indicating a progressive downward positioning of the tongue. In the second group, the IMAC maintained the same value of approximately 2 mL.

**Table 2:** In-mouth air cavity (IMAC) volume at four time points after swallowing in the two experiments.

	Mean volume (± SEM) (ml) at			
	1 second	3 seconds	9 seconds	27 seconds
Experiment 1				
Group 1 (n = 5)	6.82±2.37	13.66±3.42	18.10±3.04	22.82±3.04
Group 2 (n = 7)	2.17±0.46	1.86±0.49	2.12±0.71	2.87±0.82
Total (n = 12)	4.11±1.68	6.77±2.78	8.77±3.05	11.18±3.51
Experiment 2				
Group L (n = 13)	4.96±1.37	12.14±2.37	13.63±2.27	14.53±1.9
Group H (n = 12)	3.11±1.07	5.41±1.75	5.28±1.67	6.31±1.7
Total (n = 25)	4,03±0.87	8.91±1.6	9.62±1.6	10.59±1.52

In both experiments, the IMACs of the two subject groups were significantly different (ANOVA, SNK), although the groups were formed *a posteriori* in Experiment 1 and *a priori* on the basis of aroma release in Experiment 2. Note that the mean IMAC was similar in the two experiments.

### Experiment 2

**Figure 5:**
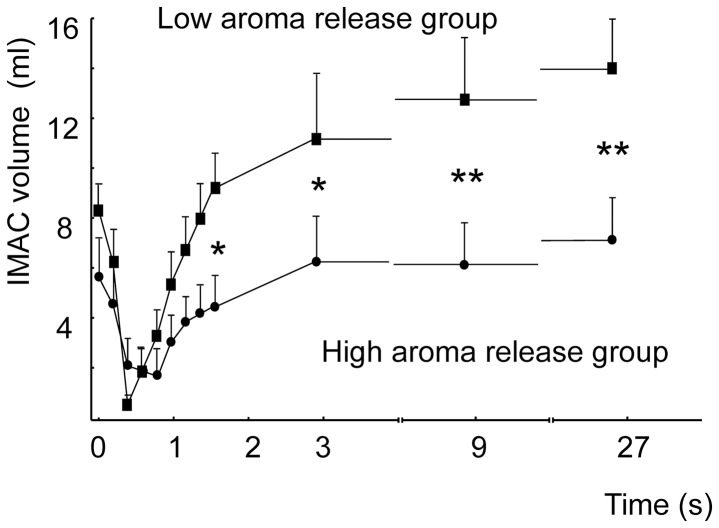
Time course of in-mouth air cavity volume during and after swallowing. Changes in the in-mouth air cavity volume (cm^3^) were measured in the two subject groups characterized by different aroma release profiles (high or low) in Experiment 2 (Closed circle: Group H; closed square: Group L). Significant differences between the two groups are indicated by an asterisk; *: P<0.05; **: P<0.01. Values (mL) are expressed as the mean ± standard error.

The IMAC volumes measured 0, 0.2, 0.4, 0.6, 0.8, 1, 1.2, 1.4, 1.6, 3, 9 and 27 seconds after swallowing was triggered are shown in Figure 5. The mean IMAC volumes of the two groups of subjects were significantly different [repeated ANOVA, F(1,22)  = 6.03; P = 0.02] and had a time effect [F(11,242)  = 12.6; P<0.001] and an interaction group*time effect [F(11,242)  = 3.75; P<0.001]. Comparisons of the two groups at the different times using MANOVA indicated a significant difference at 1.6 s [F(1,22)  = 5.89; *P* = 0.02], 3 s [F(1,22)  = 5.32; *P* = 0.03], 9 s [F(1,22)  = 8.72; *P* = 0.007] and 27 s [F(1,22)  = 9.19; *P* = 0.006]. For other times, although the low-aroma-release group came close to a null IMAC upon deglutition, its values did not differ significantly from the high-aroma-release group at other time points.

**Figure 6:**
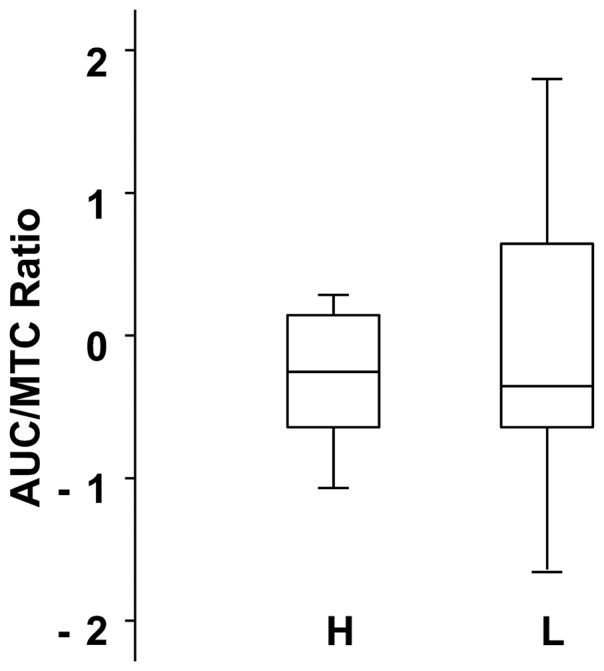
Box-plot representing the dispersion of the quantity of aroma release (AUC/MTC ratio) for the 2 groups (H and L). The box outline represents the lower quartile and upper quartile, the outermost points correspond to the smallest and largest observations, and the horizontal line is the median value. Standardization allowed the representation of the individual dispersion by equalizing the means. Standardization data consisted of subtracting the population mean from each individual's quantity of aroma release value and then dividing the difference by the population standard deviation.

The non-parametric test (Mann-Whitney U) confirmed the homogeneity of the amount of the mint tablet that the 25 subjects consumed [*Z* = 1.06; *P* = 0.28]. It also showed a significant difference between the two groups in terms of the mean quantity of aroma release [*Z* = 4.21; *P*<0.001]. In addition, the dispersion of aroma release per unit of mint tablet consumed was very different between the two groups. This was proven after the AUC/MTC data were standardized by subtracting a group's mean AUC/MTC ratio from that of each individual within the group. Individual variations were then obtained by dividing the previous difference by the population standard deviation in each group. This comparison showed greater dispersion of the population within the L group than within the H group (Figure 6). No differences were found between the two groups for sex, age, salivary flows (rSF and sSF) or respiratory flow (P>0.05, Mann-Whitney U) (Table 3).

**Table 3:** List of variables characterizing the 2 groups of subjects in Experiment 2.

Variables	H Group N = 12	L Group N = 13
AUC/MTC Ratio	4400±650	875±84
Age (years)	40.4±3.4	45.2±2.7
Sex	4F/8M	5F/8M
rSF (ml/min)	0.47±0.16	0.51±0.23
sSF (ml/min)	2.37±0.37	2.13±0.38
Respiratory Flow (L/s)	0.20±0.011	0.21±0.013

H: High-in-nose aroma release group; L: Low-in-nose aroma release group. The variable “ratio” corresponds to the amount of aroma (L-menthone) release in the nose (AUC), quantified as described in the Materials and Methods section and divided by the portion of the mint tablet the subject consumed (MTC). rSF: resting salivary flow. sSF: stimulated salivary flow. F: female, M: male. The only significant difference between the H and L groups was for AUC/MTC ratio (Mann and Whitney U). Values are expressed ± standard error.

### Comparing Experiments 1 and 2

The IMAC values measured 1, 3, 9 and 27 seconds after swallowing in the two experiments are shown in Table 2, which indicates that the means of the total values were similar in the two experiments. A progressive IMAC increase with time was observed in Group 1 of Experiment 1 and was also noted in Group L during Experiment 2. Similarly, a stable IMAC was observed in Group 2 of Experiment 1 and Group H of Experiment 2.

## Discussion

### Convergence between the two experiments

The result of this study confirmed the tested hypothesis: the type of motor events performed during empty deglutition strongly correlates with individual retro-nasal aroma release. This was inferred after the use of two methodological approaches. The rationale used for subject selection differed radically between the two experiments of this study. In the first experiment, 12 subjects were selected to form a homogenous group. Despite common physiological and demographic characteristics, the subjects in this first experiment displayed two contrasting swallowing praxis types. In contrast, subject selection in the second experiment was designed to produce a significant difference in aroma release between groups. In this second experiment, 25 subjects were selected from a pool of 68. These 25 subjects consumed a similar amount of degraded mint tablet by mouth, but two contrasted groups were constituted based on their amount of retro-nasal flavor release. These two aroma release profile groups correlated strongly with the two contrasting swallowing praxis types described in the first experiment. The convergence observed between the results of these two experiments greatly strengthens the study conclusion, i.e. retro-nasal aroma release correlates with swallowing praxis types.

### Relationship between IMAC and aroma release

These experiments indicate that two groups of subjects can be delineated on the basis of different motor patterns at work during empty deglutition. These patterns are strongly correlated with specific profiles of retro-nasal aroma release. The high-aroma-release group of Experiment 2 (Group H) displayed a small, constant IMAC after empty deglutition that corresponded to a tongue position near the palate. It is likely that for Group H, the menthone release was predominantly continuous during oral movements. The constant IMAC and the continuous aroma release profile imply a tongue-velum praxis that corresponds to a slightly but constantly opened isthmus throughout the process of chewing/swallowing. By contrast, the low-aroma-release group of Experiment 2 (Group L) released menthone primarily after a swallowing event and displayed a large IMAC following empty deglutition. The association between the large change in IMAC volume and a transient aroma release implies that the tongue-velum praxis at swallowing corresponds to a short isthmus opening preceded and followed by a steady closing of the fauces. Therefore, the vertical movements of the tongue starting at and returning to a low tongue position are associated with simultaneous velar activity that contacts the tongue and closes the fauces.

The low aroma release and a large IMAC, together with a complete closing of the isthmus before and after deglutition, can be explained by the vital need to control the food bolus while it crosses the airway. During the mastication-swallowing process, the food bolus may lie simultaneously in the mouth and the hypopharynx, which increases the potential for aspiration [25]. The bolus's movement across the fauces and its accumulation behind them, sometimes for up to 10 seconds before swallowing, has been described elsewhere [13]. A similar effect was observed with liquids [17]. The airways might be protected by the coordinated activity of the velum and the tongue, which accurately guides and controls the bolus. However, food bolus control is easy as long as the anterior velum insertion is flat. With a hollow palate, the food bolus can only be controlled by a firm closure between the tongue and velum, which requires the total closure of the isthmus during mastication and a temporal separation between mastication and deglutition. In a recent anatomical study of the IMAC at rest, two groups of subjects were described. One group displayed a high tongue position, in which the tongue appeared to rest on the palate; in the other group, the tongue rested in a low position. Interestingly, the group that had a low resting tongue position and a larger IMAC presented palates that were more hollow on average than those of the group with a high tongue position and a smaller IMAC [26].

Is the tongue-velum praxis observed during empty deglutition associated with the tongue-velum praxis during the deglutition of solid foods? The lack of intra-individual variability in the time course of the IMAC after empty deglutition suggests that there is a single individual praxis for deglutition and velum behavior. Another study reported similarities in the retro-nasal aroma release profile when subjects swallowed solid or liquid foods and found that each subject retained his or her individual retro-nasal aroma release profile regardless of the type of food consumed [6].

It is also interesting to consider the dispersion of the subjects in the different groups in terms of AUC/MTC ratio. For Group H, the dispersion of the ratio was low. As mentioned previously, the aroma release from food to the nose was continuous, with little interference from other possible physiological variables. For Group L, the dispersion was much greater, possibly due to the high IMAC and other phenomena occurring between the time the aroma was trapped in the mouth and the final swallow. It has previously been shown that equilibration of the aroma between the food material, saliva and the air in the mouth cavity during chewing does not occur immediately after the food is introduced into the mouth [27]. The equilibrium depends on the mass transfer of aromatic volatile compounds across the food/saliva interface and then from the saliva to the IMAC during mastication [7], [17]. The transfer of air from the mouth to the rhinopharynx during mastication also depends on volatile compounds released from a coating of food particles mixed with a thin film of saliva on the oral mucosal surface [28]. The mucus composition in the mouth, nasal cavity and throat and the type of salivary proteins may also play an important role in aroma release in the nose [27]. Finally, the duration of chewing may also contribute to aroma release differences because the number of mastication cycles associated with the velum opening increases as the mastication sequence nears its end [15].

### IMAC volume at rest

The mean volume of air contained in the mouth at rest can be deduced from the IMAC volume measured 27 seconds after empty deglutition. This volume was 11.18 and 10.59 mL in the first and second experiments, respectively. In contrast, Bourdiol et al. [26] reported a volume of 8.5 mL measured while the jaw was immobile. This value was likely underestimated, as the tongue was observed while the jaw was in the intercuspal position. In another study, IMAC values between 11.6 and 24.0 mL were inferred from recordings of the distance between the palate and the dorsal surface of the tongue (Bourdiol *et al.,* unpublished results). Hodgson et al. [10] integrated the expired nasal air volume with and without ongoing mastication. The 13-mL difference was considered to represent the IMAC. A range of 5 to 15 mL was proposed by Land [16]. It is likely that the substantial differences in the evaluation of IMAC volume reflect the mobility of mouth anatomic limits. Despite these variations, the present and previous studies indicate that a range of 10 to 18 mL should be considered for the IMAC volume.

### Methodological considerations

The acoustic pharyngometer has proven reliable for delineating the parameters of the human upper airway [29]–[32]. It has been used to evaluate tissue compliance during swallowing [33] but never to study the oral cavity volume either statically or dynamically. In this study, the subjects were first asked to breathe through their noses with their tongues against their palates, keeping their dental arches in the maximal intercuspal position. A residual volume was always found instead of the expected zero value. Future studies using the pharyngometer should allow for this dead space, which is generated by the oral end of the pharyngometer, by subtracting it from the measured values, as we did in the present study. It was also necessary to determine the posterior limit of the oral cavity. The two tasks performed with nasal or oral breathing, both without interdental arch contact, indicated a clear narrowing that corresponded to the faucial isthmus. The non-zero value measured at the isthmus was attributed to the volume of the dead space mentioned above.

### Conclusions

Our study presents a new, simple investigation technique for studying dynamic changes in oral volume after oral processing events. Considering the similarity of the results obtained in two different laboratories with different panels and experimenters, acoustic reflectometry has proven easy to use, reproducible and easily transferable. The technique can also be used to select population samples based on specific oral characteristics.

Studying changes in the IMAC volume after empty deglutition in humans revealed two groups characterized by different tongue-velum praxis during deglutition. One group displayed large IMAC changes, whereas the other displayed small IMAC changes. Interestingly, the profiles of aroma transfer to the nose cavity clearly differed between the two samples. Although other explanations may also help to account for the differing aroma release profiles, it appears that the observed differences were caused in large part by differences in the tongue-velum-praxis-related IMAC. The IMAC volume change thus appears to be a fundamental, individual physiological parameter that explains nose aroma release variations in humans.
